# Association between gestation length and lactation performance, lactation curve, calf birth weight and dystocia in Holstein dairy cows in Iran

**DOI:** 10.21451/1984-3143-AR2019-0005

**Published:** 2019-11-18

**Authors:** Hadi Atashi, Anise Asaadi

**Affiliations:** 1 Shiraz University Department of Animal Science Shiraz Iran Shiraz University, Department of Animal Science, Shiraz, Iran; 2 Shiraz University School of Veterinary Medicine Department of Clinical Science Shiraz Iran Shiraz University, School of Veterinary Medicine, Department of Clinical Science, Shiraz, Iran

**Keywords:** dairy cattle, genetic selection, gestation length, calving ease, dry period

## Abstract

In this study, 252,798 lactations on 108,077 cows in 433 herds were used to determine the association between gestation length (GL) and lactation performance, lactation curve, calf birth weight and dystocia in Holstein dairy cows in Iran. The GL averaged 278.1 ± 5.41 d, was categorized as short (SGL; at 1 SD below the population mean), average (AGL; the population mean ± 1 SD), or long (LGL; at least 1 SD above the population mean). Factors including parity, calf gender and calving season were associated with the GL. Primiparous cows with SGL had less lactation performance than those with longer GL; however, there was no difference between those with AGL and LGL. Multiparous cows with longer GL always had more partial and 305-d lactation performance. Primiparous cows with SGL produced less milk at the beginning of lactation and at the peak than those with AGL or LGL; inverse trends were found for lactation persistency, upward and downward slopes of the lactation curve. Within multiparous, a direct relationship was found between GL and the peak yield, where cows with longer GL always produced more milk at the peak. Multiparous cows with SGL produced less milk at the beginning of lactation, reached their peaks later, had higher lactation persistency and showed a lower upward slope of lactation curve than those with AGL or LGL. There was a direct relationship between GL and calf birth weight, where cows with longer GL had calves with more weight at the birth. Within primiparous, cows with SGL had the lowest and those with LGL had the highest rate of dystocia. However, multiparous cows with AGL had a lower rate of dystocia than those with SGL or LGL. Although there was a direct relationship between GL and lactation performance, intermediate GL seems optimal when considering dystocia.

## Introduction

Gestation length (GL), defined as the interval in days between breeding and calving, is closely related to the reproductive period ( Silveira et al., 2015 ). The GL and factors affecting it have been investigated extensively in dairy cows and it was confirmed that different breeds have different GL ( Silva et al., 1992 ; Johanson and Berger, 2003 ; Norman et al., 2009 ; Chud et al., 2014 ). There are a number of factors identified to impact GL such as genetics, month of conception (or calving), age and parity of the dam, calf gender, litter size (single vs. double), the number of days in milk, and the level of milk production ( Silva et al., 1992 ; Nogalski and Piwczyński, 2012 ; Tomasek et al., 2017 ). Although the GL is not included in genetic evaluations in most countries, several studies have confirmed that genetic variation for GL is large enough to change it through selection ( Chud et al., 2014 ; Silveira et al., 2015 ; Lacerda et al., 2018 ; Haile-Mariam and Pryce, 2019 ). The GL is associated with a number of economically important traits, including lactation performance, productive life, health traits, calving ease and calf mortality ( Hansen et al., 2004 ; Vieira-Neto et al., 2017 ; Haile-Mariam and Pryce, 2019 ). It has shown that longer GL is associated with higher calf birth weight and a higher rate of dystocia which can result in a higher rate of calf and cow mortality and longer postpartum interval ( Meijering 1984 ; Hansen et al., 2004 ; Chud et al., 2014 ; Jamrozik and Miller, 2014 ). Thus, although GL can be used in the genetic selection process, before making any recommendation to change GL, its possible effects on such traits should be considered ( Jenkins et al., 2016 ). Even though many studies have documented the association between GL and subsequent lactation performance in dairy cattle ( Norman et al., 2011 ; Jenkins et al., 2016 ; Vieira-Neto et al., 2017 ), the relationship between GL and lactation performance may be quantified more accurately and in more detail using mathematical models describing the lactation curve. To our knowledge, no study has investigated the association between GL with dam’s subsequent lactation curve in dairy cows. Therefore, the aim of this study was to investigate the association between GL and lactation curve in primiparous and multiparous Holstein cows in Iran. The association of GL with calf birth weight, dystocia, partial and 305-d lactation performance was also investigated.

## Methods

All procedures were carried out in accordance to the Shiraz University Guidelines for Animal Handling, and the project was approved by the Ethics Committee of Shiraz University. Data used in this study were records on Holstein cows collected from January 2005 to December 2014 by the Animal Breeding Center of Iran (Karaj, Iran). The herds evaluated were purebred Holsteins, managed under conditions similar to those used in most developed countries, and were under official performance and pedigree recording. The diet, fed as a TMR, was consisted of corn silage, alfalfa hay, barley grain, fat powder, beet pulp, and feed additives. Monthly milk recording was performed by trained technicians of the Iranian Animal Breeding Center, according to the guidelines of the International Committee for Animal Recording ( ICAR, 2011 ). The interval between two subsequent recordings ranged from 22 to 37 days. Almost all cows were milked three times daily (morning, afternoon, and night). Test-day milk record was defined as the sum of production for the 3 milkings, beginning with the morning milking of each recording day. After the estimation of 24-hour fat and protein percentages, 24-hour fat and protein yield were computed by multiplying the milk yield and the protein and fat percent collected on the recording day. The Interpolation method proposed by Sargent et al. (1968) , known as the reference method for calculating lactations, was used to estimate 305-d fat and protein yield. Farmers, upon observing parturition, subjectively assigned a calving ease score according to the degree of assistance provided. Recognized dystocia scores were as follows: 1 = no problem, 2 = slight problem, 3 = needed assistance, 4 = needed considerable force and 5 = extreme difficulty. In this study, dystocia scores of 1 or 2 were coded as easy calving, and scores of ≥ 3 were coded as difficult calving. Cows with missed birth date, calving date, breeding date, and parity number were deleted. Cows were required to have a minimum of 5 test-day records per lactation. Tests before 6 d or after 320 d were excluded. First calving age (FCA) was calculated as the difference between birth date and calving date at the first parity and restricted to the range of 540 to 1200 d. Ultimately, the dataset used to describe the lactation curve included 2,279,095 test-day records of 316,541 lactations on 137,206 cows distributed in 433 herds.

To describe the lactation curve, an incomplete gamma function proposed by Wood (1967) was used. The function was as follows: y _t_ = at ^b^ e ^−ct^ , where *y _t_*is the daily milk yield (kg/d) at DIM *t* , the variable *t* represents the length of time since calving, *e* is the Neper number, *a* is a parameter representing yield at the beginning of lactation, *b* and *c* are factors associated with the upward and downward slopes of the lactation curve, respectively. The incomplete gamma function was transformed logarithmically into a linear form as: ln(y _t_ ) = ln(a) + bln(t) –ct, and fitted to 2,279,095 test-day milk records corresponding to 316,541 lactations using a simple program written in Visual Basic (Microsoft Corp., Redmond, WA). The time at which peak lactation occurred (T _max_ ) was defined as: T _max_ = (b/c), expected maximum yield (y _max_ ) was calculated as: y _max_ = a(b/c) ^b^ e ^−b^ , lactation persistency (s) was calculated as: s = −(b + 1)ln(c), and total milk yield from the time of calving up to 100, 200, and 305 DIM was calculated as: $\text{y}=\text{a}\overset{\text{n}}{\underset{1}{\int }}{\text{t}}^{\text{b}}{\text{e}}^{-\text{ct}}\text{dt}$, where n = 100, 200, and 305, respectively.

Typical lactation curve has a positive *a* , *b* , and *c* , then a curve with negatives *a* , *b* , or *c* is considered atypical. Of 316,541 lactations, 63,743 (= 20.14%) were atypical and were excluded. Finally, 1,997,104 test-day milk records corresponding to 252,798 lactations on 108,077 cows were used to determine the association of GL with lactation performance, lactation curve, calf birth weight and the incidence of dystocia during the subsequent lactation. Gestation length was calculated as the difference between the date of conception and the subsequent calving date and restricted to the range of 250 to 290 d. According to Vieira-Neto et al. (2017) , the GL (averaged 278.1 ± 5.41 d) was categorized as short (SGL; at least 1 SD below the population mean), average (AGL; the population mean ± 1 SD), or long (LGL; at least 1 SD above the population mean). Cows were also grouped by parity: primiparous (n = 108077) and multiparous (n =144721) cows.

Statistical analyses were performed using SAS ( SAS Institute, 1999 ). The normality of distribution of GL, partial and 305-d lactation performance, lactation curve traits and calf birth weight were verified using the Kolmogorov–Smirnov test. The effect of factors on the GL was determined using a mixed linear model through the inclusion of herd-calving year combination (HY), two-way interaction of parity (primiparous vs. multiparous) and calving season, two-way interaction of parity and calf gender, covariate effect of FCA, and random effect of service sire. The effect of GL on the parameters describing lactation curve, partial and 305-d lactation performance was determined using multiple regression mixed models in PROC MIXED of SAS through the inclusion of GL in a two-way interaction with parity (primiparous vs. multiparous), fixed effect of (HYS), fixed effect of calf gender, covariate effect of FCA, and random effect of dam’s sire.

The effect of GL on calf birth weight was determined using the explained model, but the random effect of dam’s sire was replaced with the random effect of service sire. The effect of GL on calving difficulty was investigated using a multivariable logistic regression model through the maximum likelihood method of PROC GENMOD of SAS. In the model, the dependent variable, dystocia score, was 0 for easy and 1 for difficult calving and the independent variables were two-way interaction of parity and GL, herd, calving year, calving season, calf gender, FCA, covariate effect of calf birth weight in each sex category, and random effect of service sire.

## Results

### GL

The distribution of GL is presented in Table 1 . The mean (SD) gestation length was 278.1 (5.41) d. In primiparous cows, 14.21, 70.63 and 15.16% of all GL records were less than 273 (SGL group with mean = 268.6 and SD = 4.44 d), between 273 and 282 (AGL group with mean = 277.6 and SD = 2.64 d), and more than 282 d (LGL group with mean = 284.9 and SD =1.91 d), respectively. The corresponding values for multiparous cows were 9.39 (SGL group with mean = 268.2 and SD = 4.85 d), 66.77 (AGL group with mean = 278.0 and SD = 2.63 d), and 23.84% (LGL group with mean = 285.1 and SD =1.98 d), respectively ( Table 1 ).

**Table 1:** Categories, corresponding range, arithmetic means, standard deviation, and frequency distribution of individual GL in primiparous (n = 108,077) and multiparous (n = 144,721) Holstein cows.

Parity	Gestation length category 1	Range	Mean(d)	SD(d)	Frequency (no)	Percentage
Primiparous	SGL	250-272	268.6	4.44	15361	14.21
	AGL	273-282	277.6	2.64	76331	70.63
	LGL	283-290	284.9	1.91	16385	15.16
Multiparous	SGL	250-272	268.2	4.85	13585	9.39
	AGL	273-282	278.0	2.63	96627	66.77
	LGL	283-290	285.1	1.98	34509	23.84

1 Based on gestation length (GL), the cows in each parity were classified into three classes: SGL (more than 1 SD below the population mean), AGL (the population mean ± SD), and LGL (more than 1 SD above the population mean).

Factors including parity, calf gender, and calving season were significantly associated with GL. Both primiparous and multiparous cows carrying males had longer GL than those carrying females (P < 0.05). The average (±SE) GL in primiparous cows carrying males and females was 276.68 (±0.082) and 275.71 (±0.081) d, respectively. The corresponding values for multiparous cows were 277.58 (±0.036) and 276.59 (±0.034) for cows carrying males and females, respectively. In both the primiparous and multiparous cows, the average GL in spring and winter calving was longer than that in summer and autumn calving (P < 0.05). Within primiparous cows, the average GL (±SE) in spring, winter, summer and autumn calving was 276.91 (±0.08), 276.63 (±0.08), 275.97 (±0.08) and 275.28 (±0.08) d, respectively. The corresponding values for multiparous cows were 277.74 (± 0.04), 277.47 (± 0.04), 276.83 (± 0.04), and 276.30 (± 0.04) d for spring, winter, summer, and autumn calving, respectively.

### Association of GL with subsequent lactation performance

The GL influenced subsequent milk, fat, and protein yield ( Table 2 ), nevertheless, an important interaction was found between the GL category and parity. Primiparous cows with SGL produced less partial and 305-d lactation performance than those with AGL or LGL (P < 0.05), but there was no difference between those with AGL and LGL (P > 0.05). Within multiparous cows, there was a direct relationship between GL and lactation performance ( Table 2 ). The 305-d lactation performance as well as 100- and 200-d milk yield in multiparous cows with LGL was higher than in those with AGL (P < 0.05), and in cows with AGL was higher than in those with SGL (P < 0.05).

**Table 2:** Effects of gestation length on subsequent milk, fat and protein yield and calf birth weight in primiparous (n = 108,077) and multiparous (n = 144,721) Holstein cows.

Parity 1	GL 2	100-d milk 3 (Kg)	200-d milk 4 (Kg)	305-d milk 5 (Kg)	305-d fat (Kg)	305-d protein (Kg)	Calf birth weight (kg)
Primiparous	SGL	2940(6) ^b^	6002(10) ^b^	8760(16) ^b^	252.9(0.62) ^b^	248.4(0.60) ^b^	38.80(0.04) ^c^
	AGL	3020(3) ^a^	6138(05) ^a^	8946(08) ^a^	256.9(0.33) ^a^	252.2(0.35) ^a^	40.61(0.03) ^b^
	LGL	3029(5) ^a^	6159(10) ^a^	8982(16) ^a^	258.1(0.59) ^a^	252.5(0.60) ^a^	41.77(0.05) ^a^
Multiparous	SGL	3605(6) ^c^	6822(11) ^c^	9242(17) ^c^	263.5(0.65) ^c^	259.9(0.65) ^c^	40.52(0.04) ^c^
	AGL	3815(3) ^b^	7164(05) ^b^	9670(08) ^b^	268.2(0.33) ^b^	264.0(0.36) ^b^	42.89(0.02) ^b^
	LGL	3848(4) ^a^	7224(08) ^a^	9748(12) ^a^	*270.2(0.44) ^a^*	265.2(0.46) ^a^	44.57(0.03) ^a^

a, b, c = Least squares means with different superscripts (GL within parity) differ significantly (P < 0.05).

1 The cows were classified into two classes: primiparous cows (n = 108,077) and multiparous cows (n = 144,721).

2 Based on gestation length (GL), the cows in each parity were classified into three classes: SGL (more than 1 SD below the population mean), AGL (the population mean ± SD), and LGL (more than 1 SD above the population mean).

3 Total yield from calving up to DIM of 100 calculated as: $\text{ y}=\text{a}\overset{100}{\underset{1}{\int }}{\text{t}}^{\text{b}}{\text{e}}^{-\text{ct}}\text{dt}$.

4 Total yield from calving up to DIM of 200 calculated as: $\text{ y}=\text{a}\overset{200}{\underset{1}{\int }}{\text{t}}^{\text{b}}{\text{e}}^{-\text{ct}}\text{dt}$.

5 Total yield from calving up to DIM of 305 calculated as: $\text{y}=\text{a}\overset{305}{\underset{1}{\int }}{\text{t}}^{\text{b}}{\text{e}}^{-\text{ct}}\text{dt}$.

### Association of GL with lactation curve parameters

**Figure 1:**
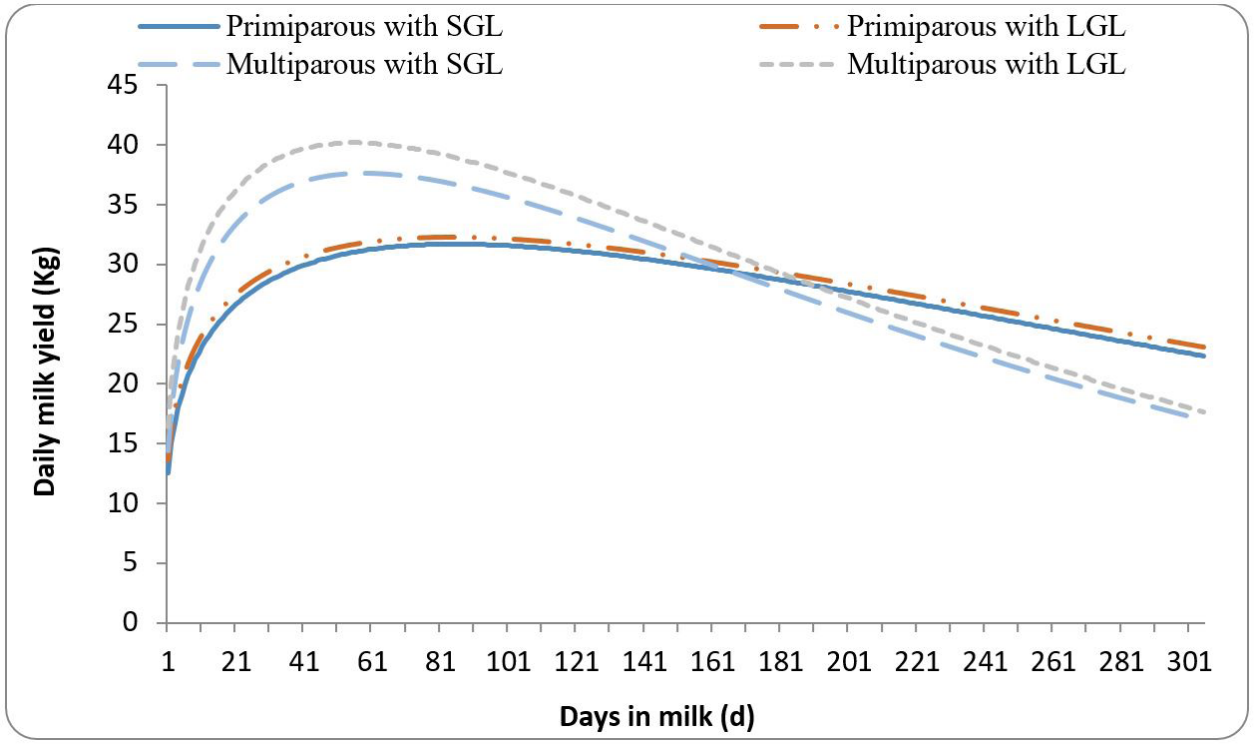
The lactation curves for primiparous and multiparous cows with SGL and LGL.

The GL affected lactation curve parameters ( Table 3 ). The lactation curves for primiparous and multiparous cows with SGL and LGL are presented in Figure 1 . Primiparous cows with SGL produced less milk at the beginning of lactation and at the peak than those with AGL or SGL (P < 0.05). However, inverse trends were found for milk yield persistency, upward and downward slopes of the lactation curve. The time at the peak was not affected by GL in primiparous cows (P > 0.05). There was no difference in lactation curve parameters between primiparous cows with AGL and LGL (P > 0.05). Multiparous cows with SGL produced less milk at the beginning of lactation, reached their peaks later, had higher lactation persistency and showed a lower upward slope of lactation curve than those with AGL or LGL (P < 0.05). However, the downward slope of the lactation curve was not affected by GL (P > 0.05). There was a direct relationship between the GL categories and the peak yield; so that multiparous cows with LGL produced more milk at the peak than those with AGL or SGL, and those with AGL had more peak yield than those with SGL (P < 0.05).

**Table 3:** Effects of gestation length on subsequent lactation curve parameters ^1^ in primiparous (n = 108,077) and multiparous (n = 144,721) Holstein cows.

Parity 2	GL 3	Ln(a) 4	b 5	c 6	s 7	Time at peak 8	Peak yield 9 (Kg/d)
Primiparous	SGL	2.53(0.006) ^b^	0.268(0.002) ^a^	0.00315(0.00003) ^a^	7.52(0.006) ^a^	103.5(1.1) ^a^	33.0(0.06) ^b^
	AGL	2.61(0.003) ^a^	0.254(0.001) ^b^	0.00306(0.00001) ^b^	7.47(0.003) ^b^	101.0(0.6) ^a^	33.6(0.03) ^a^
	LGL	2.61(0.006) ^a^	0.251(0.002) ^b^	0.00299(0.00002) ^b^	7.48(0.006) ^b^	101.0(1.0) ^a^	33.6(0.06) ^a^
Multiparous	SGL	2.67(0.007) ^b^	0.311(0.002) ^a^	0.00531(0.00003) ^a^	7.00(0.006) ^a^	61.8(1.2) ^a^	39.7(0.06) ^c^
	AGL	2.80(0.003) ^a^	0.294(0.001) ^b^	0.00528(0.00001) ^a^	6.91(0.003) ^b^	56.9(0.6) ^b^	41.8(0.03) ^b^
	LGL	2.81(0.005) ^a^	0.295(0.001) ^b^	0.00530(0.00002) ^a^	6.91(0.004) ^b^	57.0(0.8) ^b^	42.2(0.04) ^a^

a, b, c = Least squares means with different superscripts (GL within parity) differ significantly (P < 0.05).

1 Calculated using the following incomplete gamma function: ln(y _t_ ) = ln(a) + b[ln(t)] –ct, where *y _t_*is the daily milk yield (kg/d) at DIM t, the variable *t* represents the length of time since calving, *a* is a parameter representing yield at the beginning of lactation, and *b* and *c* are factors associated with the upward and downward slopes of the lactation curve, respectively.

2 The cows were classified into two classes: primiparous cows (n = 108,077) and multiparous cows (n = 144,721).

3 Based on gestation length (GL), the cows in each parity were classified into three classes: SGL (more than 1 SD below the population mean), AGL (the population mean ± SD), and LGL (more than 1 SD above the population mean).

4 Factor to represent the yield at the beginning of lactation.

5 Factors associated with the inclining slope of the lactation curve.

6 Factors associated with the declining slopes of the lactation curve.

7 Persistency, calculated as: s = −(b + 1) ln(c).

8 Time at peak calculated as: (b/c).

9 Peak yield calculated as: y _max_ = a(b/c) ^b^ e ^−b^ .

### Association of GL with calf birth weight and dystocia

The mean (SD) calf birth weight was 41.72 (5.09) kg. The least squares mean (SE) of male and female calf birth weights was 42.62 (0.02), and 40.44 (0.02) kg, respectively (P < 0.05). The GL affected calf birth weight ( Table 2 ). The average calf birth weight for cows with AGL (40.61 ± 0.03 and 42.89 ± 0.02 kg for primiparous and multiparous cows, respectively) was more than that in those with SGL (38.80 ± 0.04 and 40.52 ± 0.04 kg for primiparous and multiparous cows, respectively) and less than those with LGL (41.77 ± 0.05 and 44.57 ± 0.03 kg for primiparous and multiparous cows, respectively).

Odds ratios and corresponding 95% confidence intervals for the effects of calf gender, parity, and GL on the incidence of dystocia are presented in Table 4 . The rate of dystocia was 14.78% and was associated with the herd, calving year, calving season, calf gender, calf birth weight, and the two-way interaction of parity and GL (P< 0.05). The rate of dystocia was lower in cows delivering females than those delivering males [12.84 vs. 17.74℅, odds ratio (±95℅ CI) = 0.66 (±0.64-0.68)]. The GL influenced the rate of dystocia (P < 0.05); nevertheless, an important interaction was found between the GL category and parity. There was a linear relationship between GL and dystocia in primiparous cows; however, GL showed a nonlinear relationship with dystocia in multiparous cows ( Table 4 ). The rate of dystocia in primiparous cows with SGL was lower than in cows with AGL [19.80 vs. 20.82℅, odds ratio (±95℅ CI) = 0.88 (±0.84-0.93)], and in cows with LGL was higher than in those with AGL [22.13 vs. 20.82℅, odds ratio (±95℅ CI) = 1.20 (±1.15-1.26)]. Within multiparous, the rate of dystocia in cows with SGL (12.45%) and LGL (10.88%) was higher than that in cows with AGL (9.69%) [odds ratio (±95℅ CI) = 1.27 (±1.19-1.35) and 1.21 (±1.21-1.26) for SGL and LGL vs. AGL, respectively].

**Table 4:** Odds ratios and 95% CI for the effects of calf gender, parity 1 , and gestation length 2 on dystocia in Holstein cows (n = 252,798).

Variable	No. of births	Dystocia (℅)	Odds ratio (95 ℅ CI)	P-value
**Calf sex**				**< 0.05**
Male	100,275	17.74	Reference	
female	152,523	12.84	0.66 (0.64 – 0.68)	
**Parity × GL**				**< 0.05**
Primiparous	108,077	20.87		
SGL	15,361	19.80	0.88 (0.84 – 0.93)	
AGL	76,331	20.82	Reference	
LGL	16,385	22.13	1.20 (1.15 – 1.26)	
Multiparous	144,721	10.23		
SGL	13,585	12.45	1.27 (1.19 – 1.35)	
AGL	96,627	9.69	Reference	
LGL	34,509	10.88	1.21 (1.15 – 1.26)	

1 The cows were classified into two classes: primiparous cows (n = 108,077) and multiparous cows (n = 144,721).

2 Based on gestation length (GL), the cows in each parity were classified into three classes: SGL (more than 1 SD below the population mean), AGL (population mean ± SD), and LGL (more than 1 SD above the population mean).

## Discussion

The average GL in Holstein cows in Iran was 278.1 d, shorter than those reported by Nadarajah et al. (1989) 281.3 d, Silva et al. (1992) 280 d, and Norman et al. (2009) 281.6 d. The average GL in Danish ( Hansen et al., 2004 ) and American Holstein ( Johanson and Berger, 2003 ) was 278.5 and 277.9 d, respectively. Factors including parity of the dam, calf gender and calving season were significantly associated with GL in a close agreement with previous studies ( Nadarajah et al., 1989 ; Silva et al., 1992 ; Hansen et al., 2004 ; Nogalski and Piwczyński, 2012 ). Both primiparous and multiparous cows carrying males had longer GL than those carrying females. The average GL in cows carrying males was 1.1 ( Hansen et al., 2004 ) and 1.8 d ( Nogalski and Piwczyński, 2012 ) longer than that in those carrying females.

Primiparous cows with SGL produced less partial and 305-d lactation performance than those with longer gestation length; however, there was no difference between those with AGL and LGL. There was a direct relationship between GL and lactation performance within multiparous cows, while cows with longer GL always had more lactation performance. Vieira-Neto et al. (2017) reported that primiparous cows with AGL produced more milk than those with SGL or LGL, while multiparous cows with longer GL always produced more daily milk. Norman et al. (2011) found that multiparous cows with longer GL produced more milk, fat, and protein. However, Jenkins et al. (2016) reported that cows with either short or long GL produced less milk, fat, and protein than those with an average GL. The association between GL and lactation performance can be partly explained by this fact that the greatest increase in the mass of parenchymal tissue occurs in late pregnancy ( Davis, 2017 ); therefore, shorter the GL, less the mammary cells, and subsequently less the milk yield. In addition, it may be concluded that multiparous cows with a longer GL would have a longer dry period and more time for udder tissue recovery which can result in a higher partial and 305-d lactation performance ( Norman et al., 2011 ; Atashi et al., 2013 ). Atashi et al. (2013) reported that cows with a dry period of 51 to 60 d produced 1,212, (primiparous) and 1,166 (multiparous) kg more 305-d milk, during the next lactation than those with a dry period of ≤35 d.

Factors affecting the parameters of lactation curve have been extensively investigated in dairy cattle ( Hansen et al., 2006 ; Atashi et al., 2012 ). However, to our knowledge, this is the first report to document the association of gestation length and the parameters of the lactation curve in dairy cows. The cows with SGL had a lower yield at the beginning of lactation and higher inclining and declining slopes of the lactation curve than those with AGL or LGL. Within multiparous cows, peak yield in cows with SGL was lower than that in cows with AGL or LGL. In multiparous cows, there was a direct relationship between GL and the peak yield; longer the gestation length, more the peak yield. The average milk yield persistency in both primiparous and multiparous cows with SGL was higher than in those with AGL or LGL. Multiparous cows with a longer GL may have a longer dry period, which can result in a different lactation curve shape. Atashi et al. (2013) reported that shorter dry period (≤50 d) is associated with lower initial and peak yield, steeper inclining and declining slopes of the lactation curve, and higher milk persistency compared with a standard dry period (51-60 d).

Calf birth weight was associated with GL, whereas there was a linear relationship between GL and calf birth weight. Nogalski and Piwczyński (2012) reported a linear relationship between GL and calf birth weight; longer the gestation length, greater the birth weight of the calf. The reason is clear as two-thirds of fetal birth weight is accrued during the last trimester of gestation ( Noakes et al., 2009 ), so that fetal growth rate averages 300-400 g/d during the last month of gestation ( Meijering, 1984 ). There was a linear relationship between GL and dystocia in primiparous cows; however, GL showed a nonlinear relationship with dystocia in multiparous cows. The rate of dystocia in multiparous cows with SGL or LGL was higher than that in those with AGL. Many studies have shown a nonlinear relationship between GL and dystocia and suggested that an intermediate GL may be optimal ( Meyer et al., 2001 ; Johanson and Berger, 2003 ; Jamrozik et al., 2005 ). Vieira-Neto et al. (2017) reported that multiparous cows with either short or long GL had a higher rate of dystocia than those with intermediate GL; however, they found no association between GL categories and the rate of dystocia within primiparous cows. Nogalski and Piwczyński (2012) reported that cows with either short or long GL had a higher rate of dystocia than those with average GL. Norman et al. (2011) reported that an intermediate GL was optimal for the productive life, calving ease, stillbirth, culling, and days open. Philipsson (1976) reported that both short and long GL are associated with increased risk of dystocia. Short GL may increase dystocia through fetal mortality and premature calving, while long GL may increase the rate of dystocia through fetal oversize ( Meijering, 1984 ).

## Conclusion

This study showed that gestation length is associated with a number of economically important traits including lactation performance, lactation curve, calf birth weight, and dystocia; therefore, gestation length can be considered as an economic trait and may be incorporated into a genetic-economic index. Although there was a direct relationship between GL and lactation performance, intermediate GL seems optimal when considering dystocia. However, more studies are needed to determine optimal GL in Holstein dairy cows.

## References

[B001] Atashi H, Zamiri M, Dadpasand M (2013). Association between dry period length and lactation performance, lactation curve, calf birth weight, and dystocia in Holstein dairy cows in Iran. J Dairy Sci.

[B002] Atashi H, Zamiri MJ, Sayyadnejad MB (2012). Effect of twinning and stillbirth on the shape of lactation curve in Holstein dairy cows of Iran. Arch Tierzucht.

[B003] Chud TC, Caetano SL, Buzanskas ME, Grossi DA, Guidolin DG, Nascimento GB, Rosa JO, Lôbo RB, Munari DP (2014). Genetic analysis for gestation length, birth weight, weaning weight, and accumulated productivity in Nellore beef cattle. Livest Sci.

[B004] Davis SR (2017). Triennial lactation symposium/BOLFA: mammary growth during pregnancy and lactation and its relationship with milk yield. J Anim Sci.

[B005] Haile-Mariam M, Pryce J (2019). Genetic evaluation of gestation length and its use in managing calving patterns. J Dairy Sci.

[B006] Hansen JV, Friggens N, Højsgaard S (2006). The influence of breed and parity on milk yield, and milk yield acceleration curves. Livest Sci.

[B007] Hansen M, Lund MS, Pedersen J, Christensen L (2004). Gestation length in Danish Holsteins has weak genetic associations with stillbirth, calving difficulty, and calf size. Livest Prod Sci.

[B008] ICAR (2011). Standards and guidelines for recording milk and milk constituents. Rules and regulations.

[B009] Jamrozik J, Fatehi J, Kistemaker G, Schaeffer L (2005). Estimates of genetic parameters for Canadian Holstein female reproduction traits. J Dairy Sci.

[B010] Jamrozik J, Miller S (2014). Genetic evaluation of calving ease in Canadian Simmentals using birth weight and gestation length as correlated traits. Livest Sci.

[B011] Jenkins GM, Amer P, Stachowicz K, Meier S (2016). Phenotypic associations between gestation length and production, fertility, survival, and calf traits. J Dairy Sci.

[B012] Johanson JM, Berger P (2003). Birth weight as a predictor of calving ease and perinatal mortality in holstein cattle. J Dairy Sci.

[B013] Lacerda VV, Campos G, Roso V, Souza F, Brauner C, Boligon A (2018). Effect of mature size and body condition of Nelore females on the reproductive performance. Theriogenology.

[B014] Meijering A (1984). Dystocia and stillbirth in cattle: a review of causes, relations and implications. Livest Prod Sci.

[B015] Meyer CL, Berger P, Koehler K, Thompson J, Sattler C (2001). Phenotypic trends in incidence of stillbirth for holsteins in the United States1. J Dairy Sci.

[B016] Nadarajah K, Burnside E, Schaeffer L (1989). Factors affecting gestation length in Ontario Holsteins. Can J Anim Sci.

[B017] Noakes D, Parkinson T, England G (2009). Dystocia and other disorders associated with parturition.
*Noakes DE, Parkinson TJ, England GCW.*. Veterinary Reproduction and Obstetrics.

[B018] Nogalski Z, Piwczyński D (2012). Association of length of pregnancy with other reproductive traits in dairy cattle. Asian-Australas J Anim Sci.

[B019] Norman HD, Wright J, Kuhn M, Hubbard S, Cole J, VanRaden P (2009). Genetic and environmental factors that affect gestation length in dairy cattle. J Dairy Sci.

[B020] Norman HD, Wright J, Miller R (2011). Potential consequences of selection to change gestation length on performance of Holstein cows. J Dairy Sci.

[B021] Philipsson J (1976). Studies on calving difficulty, stillbirth and associated factors in Swedish cattle breeds: III. Genetic parameters. Acta Agriculturae Scandinavica.

[B022] Sargent F, Lytton V, Wall O (1968). Test interval method of calculating dairy herd improvement association records. J Dairy Sci.

[B023] SAS Institute (1999). SAS/STAT user’s guide.

[B024] Silva HM, Wilcox C, Thatcher W, Becker R, Morse D (1992). Factors affecting days open, gestation length, and calving interval in Florida dairy cattle. J Dairy Sci.

[B025] Silveira D, Souza F, Brauner C, Ayres D, Silveira F, Dionello N, Boligon A (2015). Body condition score of Nelore cows and its relation with mature size and gestation length. Livest Sci.

[B026] Tomasek R, Rezac P, Havlicek Z (2017). Environmental and animal factors associated with gestation length in Holstein cows and heifers in two herds in the Czech Republic. Theriogenology.

[B027] Vieira-Neto A, Galvão K, Thatcher W, Santos J (2017). Association among gestation length and health, production, and reproduction in Holstein cows and implications for their offspring. J Dairy Sci.

[B028] Wood P (1967). Algebraic model of the lactation curve in cattle. Nature.

